# Hair cortisol and adiposity in a population‐based sample of 2,527 men and women aged 54 to 87 years

**DOI:** 10.1002/oby.21733

**Published:** 2017-02-23

**Authors:** Sarah E. Jackson, Clemens Kirschbaum, Andrew Steptoe

**Affiliations:** ^1^ Department of Epidemiology and Public HealthUniversity College LondonLondonUK; ^2^ Department of PsychologyTechnische Universität DresdenDresdenGermany

## Abstract

**Objective:**

Chronic cortisol exposure is hypothesized to contribute to obesity. This study examined associations between hair cortisol concentrations, a novel indicator of long‐term cortisol exposure, and adiposity in a large population‐based sample.

**Methods:**

Data were from 2,527 men and women aged 54 and older (98% white British) participating in the English Longitudinal Study of Ageing. Hair cortisol concentrations were determined from the scalp‐nearest 2 cm hair segment, and height, weight, and waist circumference were objectively measured. Covariates included age, sex, socioeconomic status, smoking status, diabetes, and arthritis.

**Results:**

In cross‐sectional analyses, hair cortisol concentrations were positively correlated with weight (*r* = 0.102, *P* < 0.001), BMI (*r* = 0.101, *P* < 0.001), and waist circumference (*r* = 0.082, *P* = 0.001) and were significantly elevated in participants with obesity (BMI ≥30 kg/m^2^) (*F* = 6.58, *P* = 0.001) and raised waist circumference (≥102 cm in men, ≥88 cm in women) (*F* = 4.87, *P* = 0.027). Hair cortisol levels were also positively associated with the persistence of obesity (*F* = 12.70, *P* < 0.001), evaluated in retrospect over 4 years.

**Conclusions:**

Chronic exposure to elevated cortisol concentrations, assessed in hair, is associated with markers of adiposity and with the persistence of obesity over time.

## Introduction

Obesity is one of the leading risk factors for mortality worldwide, substantially increasing the risk of developing chronic diseases such as heart disease and cancers 1; thus understanding the factors that promote the onset or maintenance of obesity has important therapeutic implications. Obesity is often accompanied by a cluster of comorbidities including hypertension, impaired glucose tolerance, and dyslipidemia, known as the metabolic syndrome 2. The symptoms of the metabolic syndrome closely resemble those of Cushing's syndrome, a disorder characterized by extreme endogenous production of the stress hormone cortisol 3. Cortisol has a broad range of physiological effects throughout the human body and plays a role in glucose and lipid metabolism, body composition, and immunosuppressive and anti‐inflammatory responses 4. It is possible that long‐term hyperactivation of the hypothalamic‐pituitary‐adrenal (HPA) axis, the neuroendocrine system that regulates cortisol levels, may contribute to the development of obesity and the metabolic syndrome in otherwise healthy individuals.

Exposure to a physiological or psychological stressor activates the HPA axis, resulting in the release of cortisol. Chronic stress—and thus chronically elevated levels of cortisol—may promote obesity through effects on fat accumulation. The effects of cortisol are mediated via glucocorticoid receptors, which have a particularly high density in visceral adipose tissue. In the presence of insulin, cortisol promotes triglyceride accumulation and retention in visceral fat depots which results in increased abdominal fat 5. Indeed, animal studies have shown that chronic exposure to physical and psychological stressors increases deposition of visceral fat 6, 7.

In addition to influencing fat deposition, cortisol is associated with alterations in the quantity and type of foods consumed. In animal models, moderate and severe stressors (e.g., noise, immobilization) consistently reduce food intake, whereas mild stressors (e.g., a tail pinch) are associated with increased or unchanged intake 8. Laboratory studies in humans have shown increased *ad libitum* caloric intake in response to glucocorticoid infusion and artificially induced stress 9, 10. People tend to report eating more when stressed 11, 12, but some studies have observed reduced food intake in conditions of acute stress 13. In addition to absolute changes in energy intake, functional MRI studies have indicated that the sensitivity of central food reward circuits is lower during times of stress, which may increase cravings for “comfort foods” 14. Under stress, there is a shift in preference toward more palatable, energy‐dense foods irrespective of whether total energy intake increases 11, 12, 15. These observations are suggestive of a causal role of cortisol in the development and maintenance of obesity.

Until recently, endocrine studies have predominantly relied on cortisol assessments in saliva, urine, and blood. While these methods can be used to accurately measure momentary cortisol concentrations at the time of sampling, fluctuations in cortisol levels due to the circadian rhythm, pulsatile secretion, and daily variation arising from situational factors (e.g., environmental stressors, diet, infection 16) mean they are not well suited for capturing long‐term cortisol concentrations. Studies that have used these methods to test for associations with adiposity have yielded conflicting results and overall do not support a strong relationship between systemic cortisol and obesity 17. Over the past decade, there has been a sharp rise in studies using hair, which incorporates unbound cortisol and other lipophilic substances, as a source for cortisol analyses. Scalp hair grows at an average rate of 1 cm per month 18; thus a hair sample of 1 cm is considered to represent mean exposure to free cortisol over 1 month. Considerable evidence now supports the validity 19, 20, 21 and test–retest reliability 22 of hair cortisol concentration as a marker of long‐term cortisol exposure, as well as its robustness to a range of hair‐related factors such as dyeing and other treatments 23.

In the past 5 years, a handful of cross‐sectional studies have used hair samples to investigate the relationship between chronic cortisol exposure and adiposity, with mixed results. Several studies have reported positive associations between hair cortisol concentrations and body mass index (BMI) and waist circumference 21, 24, 25, 26, 27, 28, 29, 30, but others have not shown these correlations 21, 31, 32. Two studies have found elevated hair cortisol levels in adults with obesity relative to normal‐weight and overweight comparisons 25, 33, with similar findings observed in a large sample of children 30. In another study in adults, participants in the highest hair cortisol quartile had 2.4 times higher odds of the metabolic syndrome 27. The majority of existing studies have been conducted in relatively small samples with only one in adults and one in children having a sample >1,000, and none to our knowledge has examined hair cortisol levels in relation to adiposity using longitudinal data, which could offer greater insight into the role of cortisol in the development and maintenance of obesity.

The aim of this study was therefore to analyze associations between hair cortisol concentration and adiposity using data from a large population‐based sample over 4 years. We examined cross‐sectional associations between hair cortisol and weight, BMI, waist circumference, weight status (normal weight, overweight, obesity), and waist circumference category (raised, not raised) and looked at how hair cortisol related to the persistence of obesity over time. We hypothesized that greater and more persistent adiposity would be associated with higher hair cortisol levels.

## Method

### Study population

Data were from the English Longitudinal Study of Ageing (ELSA), a longitudinal panel study of men and women aged 50 years and older living in England. The study started in 2002, with participants recruited from an annual cross‐sectional survey of households and followed up every 2 years. The sample is periodically refreshed to ensure the full age range is maintained, and comparisons of sociodemographic characteristics with the national census indicate that the sample is broadly representative of the English population 34. The general methods of data collection are detailed at www.elsa-project.ac.uk. At each assessment, participants complete an interview and questionnaires, and in alternate (even) waves, nurse visits are conducted to obtain objective measurements of health status, including height, weight, and waist circumference. The wave 6 nurse visit included taking a hair sample to measure cortisol. The present analyses used anthropometric data from waves 4 (2008/09) and 6 (2012/13) and data on hair cortisol from wave 6. Hair analysis was carried out on a subset of the 9,169 core participants who took part in wave 6, selected at random, because of financial constraints. Data on hormone levels in hair were available for 2,685 individuals, of whom 2,601 had detectible cortisol values. We excluded 74 cases with missing data on BMI at wave 6, resulting in a final sample for analysis of 2,527 men and women. Participants gave full informed consent and ethical approval was obtained from the National Research Ethics Service.

### Hair sample collection and analysis

From all consenting participants, a lock of hair measuring at least 2 cm in length and weighing at least 10 mg was collected from the posterior vertex, cut as close to the scalp as possible. Exclusion criteria for hair sampling included pregnancy, breastfeeding, certain scalp conditions, inability to sit with head remaining still, and having less than 2 cm of hair length in the posterior vertex scalp area. Full details of the hair sampling process are provided at http://www.elsa-project.ac.uk/uploads/elsa/docs_w6/project_instructions_nurse.pdf. The wash procedure and steroid extraction were undertaken using high performance liquid chromatography–mass spectrometry, as described by Gao et al. 35. Based on an average monthly hair growth of approximately 1 cm 18, the scalp‐nearest hair segment of 2 cm represents average cortisol accumulated over an approximate time span of 2 months before sampling. Hair‐specific factors that could affect hair cortisol concentration (dyeing or chemical treatment, such as perming or chemical straightening) were assessed by self‐report.

### Measures of anthropometry

Weight was measured by nurses to the nearest 0.1 kg using portable electronic scales, and height was measured to the nearest millimeter using a portable stadiometer.

Waist circumference was measured at the midpoint between the lower rib and the upper margin of the iliac crest using a tape with an insertion buckle at one end. The measurement was taken twice, using the same tape, and was recorded to the nearest even millimeter. Those whose waist measurements differed by more than 3 cm had a third measurement taken. The mean of the two valid measurements (the two out of the three measurements that were closest to each other, if there were three measurements) was used in the analyses.

Nurses recorded any factors that might have compromised the reliability of the measurements (e.g., participant was stooped/unwilling to remove shoes), and these cases were excluded. For longitudinal analyses, we excluded cases with a weight change >10 kg if the waist circumference change was not consistent.

BMI was calculated as weight in kilograms divided by the square of height in meters and weight status defined as underweight (BMI <18.5 kg/m^2^), normal weight (BMI 18.5‐25 kg/m^2^), overweight (BMI 25‐29.9 kg/m^2^), or obesity (BMI ≥30 kg/m^2^). Raised waist circumference, an indicator of abdominal obesity, was defined according to the National Cholesterol Education Program Adult Treatment Panel III waist circumference cutoffs (≥102 cm in men, ≥88 cm in women).

### Other variables

Age, sex, and ethnicity (white/nonwhite) were included as control variables, with household nonpension wealth used as an indicator of socioeconomic status, because it has been identified as particularly relevant to health outcomes in this age group 36. Wealth was categorized into quintiles across all ELSA participants who took part in wave 6. Smoking status was assessed with the question “*Do you smoke cigarettes at all nowadays?*” (yes/no). Doctor‐diagnosed arthritis and diabetes were self‐reported in response to being presented with a list of conditions and being asked “*Has a doctor ever told you that you have (or have had) any of the conditions on this card?*”

### Statistical analysis

Analyses were performed using IBM SPSS Statistics 23. We used weights to correct for sampling probabilities and for differential nonresponse and to calibrate back to the 2011 National Census population distributions for age and sex. The weights accounted for the differential probability of being included in wave 6 of ELSA and for nonparticipation in the nurse visit. Details can be found at http://doc.ukdataservice.ac.uk/doc/5050/mrdoc/pdf/5050_elsa_w6_technical_report_v1.pdf.

Hair cortisol data were log transformed to correct skewness. For descriptive purposes, Table 1 provides information on means and standard deviations (SD) in original units (pg/mg). We used anthropometric data from wave 6 to examine partial correlations between hair cortisol levels and continuous measures of weight, BMI, and waist circumference and used ANCOVAs to analyze cross‐sectional differences in mean hair cortisol levels by weight status [normal weight, overweight, obesity (underweight participants were excluded from this analysis due to low numbers (*n* = 26)] and waist circumference status (raised, not raised). We also used longitudinal data from waves 4 and 6 to retrospectively explore associations between hair cortisol and the persistence of obesity over 4 years (never had obesity, had obesity at one wave, had obesity at both waves), using ANCOVAs to test for differences in mean hair cortisol levels between groups. All analyses were adjusted for age, sex, ethnicity, socioeconomic status (indicated by wealth quintile), hair treatment, and smoking status, as these factors have been identified in the literature as covariates for hair cortisol. We repeated the analyses adjusting for diabetes and arthritis to test for potential confounding, since these health conditions are known to be associated with both cortisol and BMI. We also performed all analyses separately by sex to explore any sex differences, but as there were no notable differences in results, we only present the whole‐sample analyses here.

**Table 1:** Sample descriptive characteristics—mean (SD) or % (*n*)

**Age (y) (*n =* 2,527)**	67.92 (7.31)
**Sex (*n =* 2,527)**	
**Male**	41.0 (1,036)
**Female**	59.0 (1,491)
**Ethnicity (*n =* 2,527)**	
**White**	98.1 (2,479)
**Nonwhite**	1.9 (48)
**Wealth quintile (*n =* 2,478)**	
**1 (poorest)**	12.6 (313)
**2**	16.6 (411)
**3**	21.8 (539)
**4**	23.4 (579)
**5 (richest)**	25.7 (636)
**Height (cm) (*n =* 2,527)**	165.49 (9.53)
**Weight (kg) (*n =* 2,527)**	77.32 (16.12)
**BMI (kg/m^2^) (*n =* 2,527)**	28.19 (5.20)
**Waist circumference (cm) (*n =* 2,512)**	95.86 (13.49)
**Weight status (*n =* 2,527)**	
**Underweight**	1.0 (26)
**Normal weight**	27.1 (685)
**Overweight**	41.4 (1,045)
**Obesity**	30.5 (771)
**Smoking status (*n =* 2,527)**	
**Current smoker**	10.0 (252)
**Non/ex‐smoker**	90.0 (2,275)
**Arthritis (*n =* 2,527)**	
**Yes**	41.1 (1,038)
**No**	58.9 (1,489)
**Diabetes (*n =* 2,527)**	
**Yes**	10.3 (261)
**No**	89.7 (2,266)
**Hair treatment (*n =* 2,521)**	
**Yes**	35.6 (900)
**No**	64.1 (1,621)
**Hair cortisol (pg/mg) (*n =* 2,527)**	30.48 (76.69)

Unweighted data from wave 6 of the English Longitudinal Study of Ageing. Numbers may not add up to the total sample number due to missing data. Valid percentages shown.

## Results

### Descriptive information about the study sample

Demographic and anthropometric characteristics of the study sample are shown in Table 1. Participants were on average 67.9 years old (range 54‐87), 59% were women, and 98% were white. Mean BMI was 28.2 kg/m^2^, 41% of participants had an overweight BMI, and a further 31% had obesity. Mean waist circumference was 96 cm (102 cm in men, 92 cm in women); 10% of participants were current smokers, 41% had arthritis, and 10% had diabetes. Just over a third (36%) of the sample reported having dyed or chemically treated hair.

### Hair cortisol and adiposity: Cross‐sectional associations

**Figure 1:**
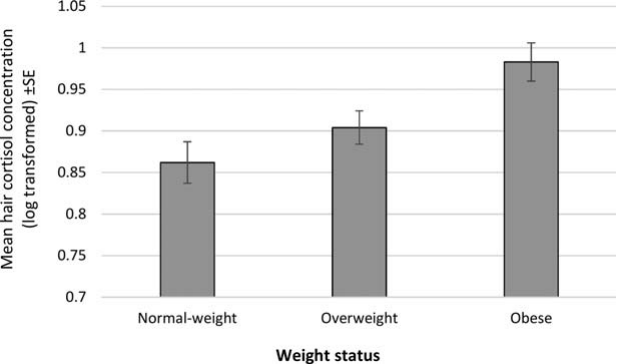
Mean hair cortisol concentrations by weight status: normal weight; (BMI 18.5‐24.9 kg/m^2^), overweight (BMI 25–29.9 kg/m^2^), obesity (BMI ≥30 kg/m^2^). Data weighted for sampling probabilities and differential nonresponse and adjusted for age, sex, ethnicity, socioeconomic status, hair treatment, and smoking status. SE, standard error.

**Figure 2:**
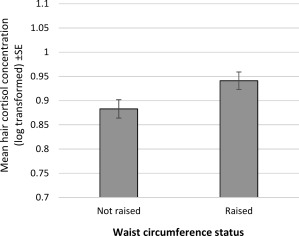
Mean hair cortisol concentrations by waist circumference status: raised (≥102 cm in men, ≥88 cm in women), not raised (<102 cm in men, <88 cm in women). Data weighted for sampling probabilities and differential nonresponse and adjusted for age, sex, ethnicity, socioeconomic status, hair treatment, and smoking status. SE, standard error.

Hair cortisol was significantly correlated with body weight (partial *r* = 0.102, *P <* 0.001), BMI (*r* = 0.101, *P <* 0.001), and waist circumference (*r* = 0.082, *P <* 0.001), with higher levels of cortisol associated with greater adiposity. There were also significant associations between hair cortisol and weight status (*F* = 6.58, *P* = 0.001) (Figure 1) and waist circumference status (*F* = 4.87, *P* = 0.027) (Figure 2). Participants who had obesity had significantly higher mean hair cortisol concentrations than normal‐weight (mean log values 0.983 vs. 0.862, *P* = 0.001) or overweight participants (0.983 vs. 0.904, *P* = 0.031). The normal‐weight and overweight groups did not differ significantly in their mean hair cortisol concentrations (0.862 vs. 0.904, *P* = 0.579). Similarly, mean hair cortisol levels were elevated in participants with a raised waist circumference compared with participants with a waist circumference in the healthy range (0.941 vs. 0.883, *P* = 0.027).

### Hair cortisol and the persistence of obesity

**Figure 3:**
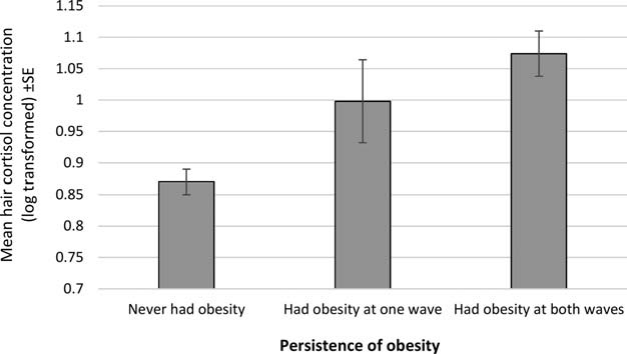
Mean hair cortisol concentrations in relation to the persistence of obesity (BMI ≥30 kg/m^2^) over a 4‐year period. Data weighted for sampling probabilities and differential nonresponse and adjusted for age, sex, ethnicity, socioeconomic status, hair treatment, and smoking status. SE, standard error.

In addition to cross‐sectional associations, hair cortisol was also retrospectively associated with the *persistence* of obesity in a subsample of participants with anthropometric data over a 4‐year period (*n* = 1,421). Mean hair cortisol increased linearly with the number of waves of obesity (BMI ≥30 kg/m^2^), from a mean log value of 0.870 in participants who did not have obesity at either time point (*n* = 1,011) to 0.998 in those who had obesity at one time point (*n* = 92) and 1.074 in those who had obesity at both time points (*n* = 318; *F* = 12.70, *P <* 0.001; Figure 3). *Post hoc* comparisons indicated a significant difference between the group who never had obesity and the group who had obesity at both times (*P <* 0.001), but differences between the group who had obesity at just one wave and the other two groups did not reach statistical significance. Within the group who had obesity at just one time point, there was no significant difference in mean hair cortisol between those who had obesity at the first wave (*n* = 47) and those who had obesity at the second wave (*n* = 45; mean log values 1.050 vs. 0.942 respectively, *P* = 0.444).

### Adjustment for associated health conditions

Hair cortisol concentrations were slightly higher in participants with diabetes (mean log values 0.991 vs. 0.907, *F* = 3.78, *P* = 0.052) but did not differ by arthritis status after adjustment for covariates (mean log values 0.926 vs. 0.910, *F* = 0.35, *P* = 0.557). Replicating our analyses with additional adjustment for patient‐reported diabetes and arthritis had little effect on the results. Cross‐sectional associations remained significant between hair cortisol levels and weight (*r* = 0.096, *P <* 0.001), BMI (*r* = 0.095, *P <* 0.001), waist circumference (*r* = 0.075, *P* = 0.001), and weight status (*F* = 5.50, *P* = 0.004). The association with waist circumference status became nonsignificant (*F* = 3.67, *P* = 0.056). The association with persistence of obesity was also significant after adjustment for arthritis and diabetes (*F* = 10.78, *P <* 0.001).

## Discussion

This study examined associations between long‐term cortisol levels, as assessed in hair, and adiposity in a large population‐based sample. In cross‐sectional analyses, hair cortisol concentrations were positively correlated with body weight, BMI, and waist circumference and were significantly elevated in participants with obesity and raised waist circumference. In retrospective longitudinal analyses, hair cortisol levels were also significantly associated with the persistence of obesity over 4 years. Results were robust to adjustment for a range of sociodemographic and health‐related variables.

Previous studies examining relations between cortisol and adiposity have largely relied on measurements of cortisol in serum, saliva, or urine, which vary according to the time of day and a range of other situational factors 16. The analysis of cortisol in scalp hair reflects systemic cortisol exposure over a prolonged period—in this study, 2 months—and is therefore not affected by the timing of sample collection or acute stress. Consequently, using hair samples to assess cortisol offers a particularly well‐suited method for investigating the effects of long‐term cortisol levels in epidemiological studies.

A few small studies have assessed cortisol in scalp hair in relation to BMI, and their results have been mixed. For example, Stalder et al. reported a strong association between hair cortisol and BMI in two independent studies (*n* = 58‐155) that remained significant even when the analysis was restricted to participants without obesity 26 and observed a similar association in a large workplace study (*n* = 1,258) 27. However, neither Manenschijn et al. 21 (*n* = 205) nor Feller et al. 28 (*n* = 654) found long‐term cortisol levels to be significantly related to BMI, despite being correlated with waist circumference. Olstad et al. 32 found no relation between BMI and hair cortisol in women (*n* = 70) or children (*n* = 30) living in disadvantaged neighborhoods, although BMI was significantly associated with perceived stress. In a much larger, nationally representative sample, we observed a significant positive correlation between BMI and hair cortisol concentration. A significant association between hair cortisol and BMI has also very recently been observed in a large sample of 6‐year‐old children (*n* = 3,019) 30. The correlation between waist circumference, an indicator of visceral fat, and long‐term cortisol has been more consistently reported across previous studies 21, 24, 27, 28, 29. We also demonstrated a significant association between a raised waist circumference, a proxy for abdominal obesity, and hair cortisol.

A handful of studies have observed an association between long‐term cortisol and obesity, as we did in this study. Wester et al. 33 found that patients with obesity (*n* = 47) being evaluated in an obesity center had significantly higher hair cortisol levels than normal‐weight (*n* = 41) and overweight controls (*n* = 87), but there was no significant difference between the normal‐weight and overweight groups. Similar results have been observed in samples of young shift and day workers 25 and children 24, 30. Our study extends previous work by using data from two time points, 4 years apart, to retrospectively assess the relation between hair cortisol and the persistence of obesity. We found a linear association between the number of waves of obesity (0, 1, or 2) and hair cortisol levels: mean cortisol was lowest in individuals who did not have obesity at either time point, higher in those who had obesity at just one time point, and highest in those who had obesity at both times. This suggests that chronic high‐level cortisol exposure may play a role in the maintenance of obesity. However, it is important to note that as BMI measurements preceded the cortisol measurement, a reverse association is also possible whereby persistent obesity could play a role in the elevation of cortisol levels, for example through altered metabolism.

Just one study to our knowledge that has examined hair cortisol in relation to multiple measures of adiposity has failed to find any evidence of an association 31. The authors attributed the null findings to the elderly sample (*n* = 283), given known changes in body fat distribution with aging 37. However, in a more recent, larger study of middle‐aged and older adults (*n* = 654), waist circumference and waist‐to‐hip ratio (but not BMI) were significantly associated with hair cortisol concentrations 28. This study's sample also comprised middle‐aged and older adults and we observed significant relations between hair cortisol and all markers of adiposity. Whether the associations between cortisol and adiposity differ by age is an issue that requires further exploration.

This study has several strengths. Data were from a large, well‐characterized, nationally representative sample of middle‐aged and older men and women. This is an advantage over previous studies that have used smaller convenience samples. We used anthropometric data that were objectively measured by trained research nurses rather than relying on self‐reports. The mean BMI and prevalence of overweight and obesity in our sample were comparable to age‐matched estimates from the 2012 Health Survey for England 38. Availability of data on BMI at multiple time points allowed for analysis of hair cortisol in relation to the persistence of obesity over time, which to our knowledge has not previously been done. We controlled for a range of potential behavioral and health‐related confounders.

There were also limitations. Data were from an older population, in which levels of cortisol may differ relative to younger adults 28, and the sample was almost exclusively white, so findings cannot be assumed to generalize. Data on cortisol were only available for a subset of the full ELSA sample, but the sample that provided hair samples was selected at random. Our analyses did not account for use of medications that might influence cortisol concentration, such as oral, intra‐articular, or local glucocorticoids. A key limitation is that hair samples were only collected in the most recent health examination. This is because the sampling of cortisol in hair was not widely known in 2008 when the previous health examination was conducted. It is not currently known whether chronically elevated cortisol levels are a cause or consequence of obesity, and since it was not possible to analyze anthropometric changes over time in relation to baseline hair cortisol level in this study, we were not able to draw conclusions on causality. Longitudinal studies are required to shed light on the direction of associations between cortisol and adiposity. However, evidence from the clinical literature showing that extreme conditions of hypercortisolism (Cushing's syndrome) and hypocortisolism (Addison's disease) lead to central obesity and weight loss, respectively 39, suggests that increased systemic cortisol exposure may be the driving force behind this association. If this is found to be the case, targeting cortisol levels may offer a novel method for treating obesity 40.

To conclude, these results provide consistent evidence that long‐term exposure to elevated levels of cortisol over several months is associated with higher levels of adiposity. Hair cortisol offers a suitable and easily obtainable measure for assessing chronically elevated cortisol concentrations in obesity research and may therefore aid in further advancing understanding in this area. While cross‐sectional studies have provided a good starting point from which to explore the role of HPA axis dysregulation and chronic cortisol exposure in the development of obesity, longitudinal research is needed in order to clarify the direction of associations.
